# Sub-Cellular Localization of Metalloproteinases in Megakaryocytes

**DOI:** 10.3390/cells7070080

**Published:** 2018-07-20

**Authors:** Alessandro Malara, Daniela Ligi, Christian A. Di Buduo, Ferdinando Mannello, Alessandra Balduini

**Affiliations:** 1 Department of Molecular Medicine, University of Pavia, 27100 Pavia, Italy; alessandro.malara@unipv.it (A.M.); christian.dibuduo@unipv.it (C.A.D.B.); 2 Laboratory of Biotechnology, IRCCS San Matteo Foundation, 27100 Pavia, Italy; 3 Section of Clinical Biochemistry and Molecular Genetics, Department of Biomolecular Sciences, University “Carlo Bo” of Urbino, 61029 Urbino, Italy; daniela.ligi@uniurb.it (D.L.); ferdinando.mannello@uniurb.it (F.M.); 4 Department of Biomedical Engineering, Tufts University, Medford, MA 02155, USA

**Keywords:** megakaryocyte, metalloproteinase, thrombopoiesis

## Abstract

Metalloproteinases (MMPs) are zinc-dependent endopeptidases that play essential roles as the mediator of matrix degradation and remodeling during organogenesis, wound healing and angiogenesis. Although MMPs were originally identified as matrixin proteases that act in the extracellular matrix, more recent research has identified members of the MMP family in unusual locations within the cells, exerting distinct functions in addition to their established role as extracellular proteases. During thrombopoiesis, megakaryocytes (Mks) sort MMPs to nascent platelets through pseudopodial-like structure known as proplatelets. Previous studies identified gelatinases, MMP-2 and MMP-9, as a novel regulator system of Mks and the platelet function. In this work we have exploited a sensitive immunoassay to detect and quantify multiple MMP proteins and their localization, in conditioned medium and sub-cellular fractions of primary human CD34^+^-derived Mks. We provide evidence that Mks express other MMPs in addition to gelatinases MMP-2 and MMP-9, peculiar isoforms of MMP-9 and MMPs with a novel nuclear compartmentalization.

## 1. Introduction

The bone marrow (BM) environment is composed of various types of cells surrounded by a meshwork of their secreted extracellular matrix (ECM) components [1]. The turnover of ECM is fundamental for the structural and functional homeostasis of BM hematopoiesis. The importance of ECM in physiologic hematopoiesis and its pathologic modifications in hematopoietic malignancies are becoming evident and are under extensive investigation [2]. Megakaryocytes (Mks) are rare cells in the BM and, besides releasing platelets, they participate in the establishment and maintenance of the BM cell niche in both physiologic and pathologic conditions [3,4,5,6]. Interestingly, Mks are involved in ECM deposition and remodeling [7], as demonstrated by their role in fibronectin (FN) fibrillogenesis [8] and the expression of ECM structure modifiers, such as lysyl oxidase and factor XIIIa, essential to the dynamic of Mk-ECM component interactions [8,9].

During thrombopoiesis, Mks sort metalloproteinases (MMPs) to nascent platelets through a pseudopodial-like structure known as proplatelets [10]. MMPs are zinc-dependent endopeptidases that play essential roles as the mediator of matrix degradation and remodeling during stem cell differentiation, organogenesis, wound healing and angiogenesis [11,12,13]. MMP family proteins are divided in five groups by their respective substrates or cellular localization: Stromelysins (e.g., MMP-3, MMP-10, MMP-11); matrilysins (e.g., MMP-7); collagenases (MMP-1, MMP-8, MMP-13, MMP-18); gelatinases (MMP-2 and MMP-9); membrane-type (MT-MMPs) and other MMPs (3).

Previous studies identified the gelatinases MMP-2 and MMP-9 as forming a novel regulator system of Mks and the platelet function. Induction of MMP-9 expression, but not MMP-2, by the chemotactic activity of Stromal Derived Factor-1 (SDF-1), is considered a key step in modulating Mk migration through the basement membrane of BM sinusoids and subsequent platelet release [14]. In resting platelets, MMP-2 is randomly distributed, not associated with platelet granules and is released upon platelet stimulation to regulate platelet activation and aggregation in physiological hemostasis or pathophysiological formation of occlusive thrombi [15,16,17]. The presence and activity of MMP-9 and its isoforms in platelets is still debated [18,19]. Aside from gelatinases, other MMPs have been detected in Mks and platelets by different groups using multiple techniques, including immunofluorescence, western blot, next generation RNA sequencing or PCR analysis [20,21]. Cecchetti et al. identified transcripts for MMP-1, 11, 14, 15, 17, 19, 24 and 25 by performing a RNAseq screen of MMP expression in primary human Mks, while expression of MMP-3 has still to be clarified [20,21].

Although MMPs were originally identified as proteases with a peculiar function in the extracellular matrix, more recent research has identified members of the MMP family in unusual locations within the cells, exerting distinct functions in addition to their established role as extracellular proteases [22]. To this regard, MMPs have been detected in the cytosol, organelles and extracellular compartments and more recently several types of MMPs were found in the nucleus [23,24,25] Nuclear MMPs are supposed to cleave nuclear matrix proteins, although other possible functions are beginning to emerge [26]. To this regard, MMP-1, MMP-2, MMP-9 and MMP-13 in cell nuclei of brain neurons, endothelial cells and cardiac myocytes are supposed to regulate the activity of proteins involved in DNA repair and apoptosis [27,28,29]. MMP-3 has an unprecedented role as a transcription factor that is independent of its enzymatic activity [30]. Further, new intracellular roles such as the cleavage of intracellular non-matrix proteins, activation/inactivation of intracellular substrates and signal transduction are increasing the functional plasticity of these enzymes.

Thus, following the emergence of these untraditional functions of MMPs in the extracellular space, as well as, in the cytosol and nucleus, we have performed a sensitive immunoassay to detect multiple MMP proteins and their localization, in conditioned medium and sub-cellular fractions of primary human Mks.

## 2. Material and Methods

### 2.1. Cell Culture

Human cord blood was collected following normal pregnancies and deliveries upon the informed consent of the parents. All samples were processed in accordance with the ethical committee of the IRCCS Policlinico San Matteo Foundation and the principles of the Declaration of Helsinki. CD34^+^ hematopoietic were purified by immunomagnetic beads selection and cultured in StemSpan medium (Stem Cell Technologies, Vancouver, BC, Canada) supplemented with 1% L-glutamine, 1% penicillin-streptomycin, 10 ng/mL of human recombinant Thrombopoietin (TPO) and Interleukin-11 (IL-11) (PeproTech, London, UK), at 37 °C in a 5% CO_2_ fully humidified atmosphere for 13 days as previously described [8]. Medium was changed at day 3, 7 and 10 of differentiation.

### 2.2. Flow Cytometry

Purity of Mk culture, at day 13 of differentiation, was analyzed by staining cells with FITC anti-human CD41 (clone HIP-8) and PE anti-human CD42b (clone HIP-1) antibodies (all from Biolegend, Milan, Italy). Samples were acquired with a Beckman Coulter FacsDiva flow cytometer (Beckman Coulter Inc., Milan, Italy). Relative isotype controls were used to set the correct analytical gating. FITC mouse IgG (clone MOPC-21) and PE-mouse IgG (clone MOPC-21), isotype controls were purchased from Biolegend (Milan, Italy). Off-line data analysis was performed using Beckman Coulter Kaluza^®^ version software package.

### 2.3. Real Time PCR

Retrotranscription (RT) was performed using the iScriptTM cDNA Synthesis Kit according to manufacturer instructions (BioRad Laboratories Inc., Milan, Italy). For quantitative Real Time PCR, RT samples were diluted 1:3 with ddH2O and the resulting cDNA was amplified in triplicate in reaction mixture with 200 nM of each specific primer and SsoFast™ Evagreen^®^ Supermix (Bio-rad Laboratories, Milan, Italy). The amplification reaction was performed in a CFX Real-time system (BioRad Laboratories Inc., Milan, Italy) with the following protocol: 95 °C for 5 min, followed by 35 cycles at 95 °C for 10 s, annealing at 60 °C for 15 s, extension at 72 °C for 20 s. Pre-designated KiCqStart™ primers for MMP1, MMP2, MMP3, MMP7, MMP8, MMP9, MMP10, MMP12, MMP13 and GAPDH genes were purchased from Sigma-Aldrich (Milan, Italy). The BioRad CFX Manager^®^ software 3.0 was used for quantitative analysis (BioRad Laboratories Inc., Milan, Italy).

### 2.4. Zymography

Aliquots of all samples were analyzed by gelatin zymography carried out on 6.5% polyacrylamide gels copolymerized with 3 g/L 90 Bloom Type A gelatin from porcine skin (from Sigma-Aldrich, Milan, Italy). Samples were loaded native with the addition of SDS zymogram sample buffer (62.5 mM Tris-HCl, pH 6.8, 25% glycerol, 4% SDS, 0.01% bromophenol blue). SDS-PAGE gels were run using a Bio-Rad Mini-Protean III apparatus (Bio-Rad, Hercules, CA, USA) in SDS running buffer (25 mM Tris, 192 mM glycine, and 0.1% *w*/*v* SDS) at a constant voltage of 105 V. After electrophoresis, gels were incubated for 40 min at room temperature on a rotary shaker in Triton X-100 2.5%, to remove SDS. The gels were washed with distilled water and incubated for 24 h in enzyme incubation buffer (containing 50 mM Tris; 5 mM CaCl2; 100 mM NaCl; 1 mM ZnCl2; 0.3 mM NaN3, 0.2 g/L of Brij^®^-35; and 2.5% *v*/*v* of Triton X-100, pH 7.6) at 37 °C. Staining was performed using Coomassie Brilliant Blue R-250 (0.2% *w*/*v* Coomassie brilliant blue in 50% *v*/*v* methanol and 20% *v*/*v* acetic acid). Gels were destained with a destaining solution (50% *v*/*v* methanol and 20% *v*/*v* acetic acid) until clear gelatinolytic bands appeared against the uniform dark-blue background of undigested protein substrate. Gelatinase calibrators (as molecular weight standards) were prepared by diluting healthy capillary blood with 15 volumes of non-reducing Laemmli sample buffer. It is important to specify that whole capillary blood, used as a calibrator, presents only the zymogens of gelatinases: pro-MMP- 2 at 72 kDa, pro-MMP-9 at 92 kDa, and pro-MMP-9 complexes at 130 kDa (MMP-9/NGAL), and 225 kDa (MMP-9 multimeric form) as previously recognized by monoclonal anti-MMP-2 and anti-MMP-9 antibodies and characterized as latent pro-enzymes, activated by APMA and inhibited by both calcium and zinc chelators (EDTA and o-phenanthroline, respectively). Zymographic bands were densitometrically measured with the image analyzer LabImage 1D (Kapelan, Leipzig, Germany) [31].

### 2.5. Cell Fractionation

Megakaryocytes at day 13 of differentiation were collected, centrifuged and washed with PBS twice. At least 2 × 10^6^ cells per experiments were used. The subcellular fractionation was performed according to the REAP method [32]. Briefly, the cells were collected in 1.5 mL microcentrifuge tubes in 1 mL of ice-cold PBS. After centrifugation (a popo-spin for 10 s in an Eppendorf table top microfuge), supernatants were removed from each sample and cell pellets were re-suspended in 900 µL of ice-cold 0.1% NP-40 detergent in PBS and triturated 5-times using a p1000 micropipette (whole lysate). An aliquot of 600 µL of the whole lysate was centrifuged for 10 s in 1.5 mL microcentrifuge tubes and then the supernatant was removed as the cytosolic fraction. The pellet was re-suspended in 0.5 mL of ice-cold 0.1% NP-40 detergent in PBS and centrifuged as above for 10 s and the supernatant was discarded. The pellet was re-suspended in 200 µL of native Laemmli sample buffer containing DNAse I (0.01U/µL), triturated 5-times using a p200 micropipette, designated as nuclear fraction and used for both quantitative MMP assays and qualitative gelatinase zymography.

### 2.6. MMPs Multiplex Array

Quantification of MMPs was performed at day 13 of differentiation from 1 × 10^6^ cells, prior to in viro platelets release, to avoid the release of intracellular proteins into conditioned supernatants as a consequence of the apoptotic-like process of proplatelet formation. Supernatants were collected, pre-cleared by centrifugation at 15,600 g at 4 °C for 20 min and stored at −80 °C or immediately analyzed. MMP concentrations in all samples (supernatants and subcellular fractions from at least seven cultures) were quantified through the Pro™ Human MMP 9-plex Assay (including: MMP-1, MMP-2, MMP-3, MMP-7, MMP-8, MMP-9, MMP-10, MMP-12, MMP-13). The assay was based on multiplex suspension immunomagnetic method using fluorescently dyed magnetic beads covalently conjugated with monoclonal antibodies (Bioplex, BioRad Labs, Hercules, CA, USA).

To avoid subcellular ‘matrix’ artifacts during assay caused by interfering substances in culture media, we serially diluted randomly selected serum-free media, reanalyzing them for the response linearity. The lower detection limit for all MMPs was 1.0 pg/mL, and the mean intra-assay variability was 10%.

Concentrations of all MMPs were determined using a Bio-Plex 200 system, based on Luminex X-Map Technology (BioRad Labs, Hercules, CA, USA). Data were analyzed using BioManager analysis software (version 6.1). The protein concentrations (expressed as pg/mL) were calculated through a standard curve [33].

### 2.7. Immunofluorescence

Megakaryocytes at day 13 of differentiation were cytospun onto Poly-L-Lysine-coated coverslips. Cells were fixed with PFA 4% and permeabilized with Triton 0.5%. Cells were stained with anti MMP-2 antibody (Abcam, Cambridge, UK, catalog number AB37150) diluted 1:200 overnight at 4 °C. Alexa 594-conjugated secondary antibody was purchased from Invitrogen (Milan, Italy). Nuclei were counterstained using Hoechst 33258 (100 ng/mL in PBS) at room temperature. Slides were then mounted with micro-cover glasses using Prolong Antifade Reagent (Invitrogen, Milan, Italy). Negative control was performed by omitting the primary antibody. Images were acquired with a TCS SP5 II confocal laser scanning microscope (Leica, Heidelberg, Germany).

## 3. Results and Discussion

**Figure 1:**
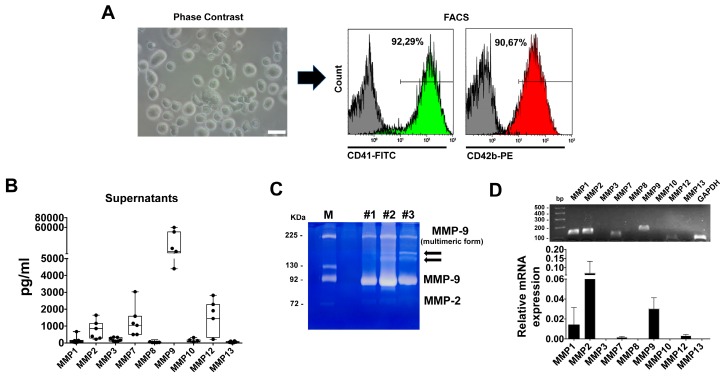
Expression profiling of MMPs in primary human Megakaryocytes. (**A**) Phase contrast image of Megakaryocyte (Mk) culture at day 13 of differentiation. Scale bar = 50 μm. Purity was analyzed by FACS, after staining with Mk markers CD41 (Green histogram) and CD42b (Red histogram). FITC Isotype IgG and PE Isotype IgG were used to set the analytical gate (Grey histograms). (**B**) Profiling and quantification of MMPs in supernatants from 1 × 10^6^ megakaryocytes at day 13 of differentiation. N = 7. (**C**) Representative zymography of Mk supernatants at day 13 of culture. Three independent samples are shown. M = Molecular Marker. Arrows indicate potential dimers of MMP-9 activated forms (160–180 kDa). (**D**) Representative RT-PCR products of MMPs in Mks at day 13 of differentiation and relative quantification from at least three independent experiments. GAPDH expression was used for normalization.

To identify MMPs that are expressed during human thrombopoiesis, nine of 24 family members of MMPs were simultaneously analyzed (MMP-1, MMP-2, MMP-3, MMP-7, MMP-8, MMP-9, MMP-10, MMP-12, MMP-13) by means of a Multiplex Array. We focused on mature Mks at day 13 of differentiation to define the MMP repertoire that these cells harbor prior to platelet release. Mk purity was verified by FACS analysis after CD41 and CD42b staining. Only cell cultures that displayed a fraction of double positive CD41/CD42b cells higher than 90% by flow cytometry were then subjected to biochemical analysis of MMP content and zymography (Figure 1A). Analysis of fresh medium gave negative results for all the analytes (Data Not Shown).

Quantitative assessment of conditioned medium revealed that the MMP-9 protein was the most abundant in conditioned medium in agreement with its previous described role as the regulator of Mk function in the extracellular space (mean 4396 pg/mL from 1 × 10^6^ cells) [14]. Significant amounts of MMP-2 (mean 1162 pg/mL), MMP-7 (mean 1045 pg/mL) and MMP-12 (mean 2287 pg/mL) were also detected (Figure 1B). Interestingly, the presence and activity of MMP-2 and MMP-9 can be readily appreciated by in gelatin zymography analysis of Mks conditioned medium at day 13 of culture (Figure 1C), and to the best of our knowledge, this is the first report that identifies all the isoforms of MMP-9. In particular, consistent gelatinolytic bands were easily identified at 82–86 kDa (as activated forms), and at 225 kDa (as complexed form). Interestingly, for the first time, we identified in the conditioned medium of Mks at day 13 of culture two further gelatinolytic bands at the range 160–180 kDa, probably dimers of MMP-9 activated forms (i.e., devoid of pro-domains) and unusual complexes of MMP9 with Neutrophil Gelatinase Associated-Lipocalin complexes (NGAL), which protects MMP-9 from proteolytic degradation and enhances its enzymatic activities [34]. Our evidence is in agreement with the crucial roles of MMPs (in particular of both gelatinases) in platelet functions, shedding further lights on the enzymatic activity of activated MMP-9 forms during the differentiation of Mks [35]. Notably, MMP-7 and MMP-12 may represent potential released products during thrombopoiesis. MMP-7 is capable of degrading a wide array of ECM components such as gelatin, fibronectin, laminin and elastin. Further, MMP-7 can cleave the pro-domain of the gelatinases MMP-2 and -9 [36], but its relevance under physiological conditions is still debated. To date, MMP-7 has been implicated in several physiological processes, such as wound healing, innate immunity and cancer [37,38]. MMP-12 however, is recognized as a macrophage-secreting proteinase. MMP-12 myeloid-restricted over-expression in mice has a significant impact on the development, differentiation and commitment of hematopoietic progenitor cells to myeloid lineage cells in the BM [39]. Thus, while MMP-12 may act as a pleiotrophic molecule with roles in hematopoiesis and myelopoiesis, involvement of MMP-7 in Mk biology is far from being fully understood. While MMP-7 has never been detected in platelets, Wang et al. recently reported on the expression of MMP-12 in platelets [40].

On the contrary, very low levels of MMP-1, MMP-3, MMP-8, MMP-10 and MMP-13 were detected in culture conditioned medium (Figure 1B). RT-PCR was then applied to screen for MMP mRNAs that are expressed during human thrombopoiesis. As shown in Figure 1D, gene expression of MMPs, at day 13 of differentiation, confirmed a prevalence of MMP-1, MMP-2 and MMP-9 transcripts and to a lesser extent of MMP-7 and MMP-12. Moreover, other MMPs were not detected by this assay.

**Figure 2:**
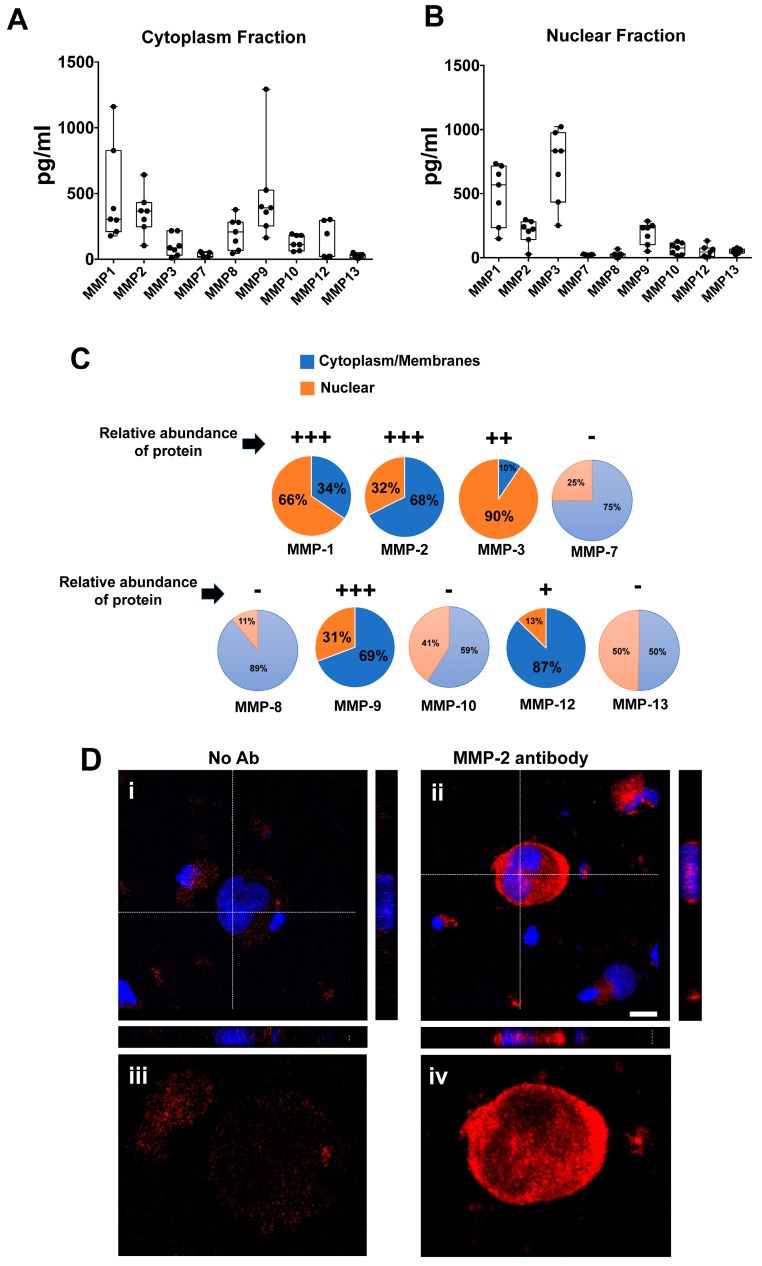
**Subcellular localization of MMPs in primary human Megakaryocytes.** (**A**,**B**) Profiling and quantification of MMPs in cytoplasm (**A**) and nuclear (**B**) fractions of 2 × 10^6^ megakaryocytes at day 13 of differentiation. N = 7. (**C**) Schematic representation of intracellular distribution of individual MMP between the cytosolic/membrane and nuclear compartments. Relative intracellular abundance is also provided (−/absent, +/low level, ++/intermediate level, +++/high level). (**D**) Immunofluorescence analysis of intracellular localization of MMP-2 in megakaryocytes at day 13 of differentiation. In the panel (**i**), the primary antibody was omitted as negative control, while Hoechst was used to highlight nuclei. In the panel (**ii**), cells were stained with an anti MMP-2 antibody and Hoechst. Orthogonal cross-sections of representative z-stack from cell nuclei are also provided. In panels (**iii**,**iv**), higher magnification of MMP-2 staining in the absence or presence of the primary antibody is provided. Scale bar = 10 µm.

Next, to measure the intracellular localization of MMPs in cultured Mks, membrane/cytosolic and nuclear fractions from seven independent cultures were prepared. At day 13 of culture, 2 × 10^6^ mature Mks were centrifuged, washed three times with PBS and subjected to cell fractionation. Quantification of MMPs by multiplex array revealed that cytosolic fractions displayed appreciable amounts of several MMPs, including MMP-1 (mean 299.18 pg/mL), MMP-2 (432 mean pg/mL), MMP-8 (mean 283 pg/mL), MMP-9 (mean 527.09 pg/mL) and MMP-12 (mean 303 pg/mL) (Figure 2A). Negligible levels or almost complete absence of MMP-3, MMP-7 and MMP-13 were detected under our experimental conditions. More importantly, detection and quantification of MMPs in nuclear extracts revealed that MMP-1 (mean 568.57 pg/mL) and MMP-3 (mean 833.42 pg/mL) were the most abundant proteins with nuclear localization. To a lesser extent also MMP-2 (206.33 pg/mL) and MMP-9 (mean 232,72 pg/mL) were detected (Figure 2B). A schematic representation of intracellular distribution of individual MMPs and relative protein abundance is provided in Figure 2C.

In addition, nuclear localization of MMPs was investigated by immunofluorescence procedures. Interestingly, analysis of MMP-2 localization, by immunofluorescence, showed a diffuse pattern in the cytoplasm as well as co-localization with Hoechst in nuclei of Mks (Figure 2D). Control cells, stained only with the secondary antibody, did not show a nuclear signal.

The few studies available addressing the function of nuclear MMPs suggest a role in apoptosis induction [26,27,29]. Nevertheless, of all the hematopoietic processes occurring in the BM, the production of Mks and, subsequently, platelets are the most complex and unusual. To this regard, the precise physiological role of the apoptotic process in Mks and platelets is yet to be established [41]. Apoptotic-like features are associated with Mk cytoplasm conversion into a mass of proplatelets, which are released from the cell. The remaining senescent and denuded Mk nuclei, after platelet release, are disposed by apoptosis and phagocytosis [42]. However, signals controlling these events are not known and, at the moment we can only speculate, about the potential involvement of nuclear MMPs in these processes.

Collectively, our data demonstrated that primary Mks expressed additional MMPs to the previously identified gelatinases, MMP-2 and MMP-9 peculiar isoforms, and revealed an unprecedented and novel/intriguing nuclear localization. To this regard, we are aware that nuclear localization of MMPs should be confirmed by means of several techniques, however the accuracy of the technology employed and the reproducibility of this novel compartmentalization of MMPs in Mks reveal novel regulatory functions. Further investigations will help in dissecting the role of the different MMPs in regulating platelet production.

## References

[1] Malara A., Currao M., Gruppi C., Celesti G., Viarengo G., Buracchi C., Laghi L., Kaplan D.L., Balduini A. (2014). Megakaryocytes contribute to the bone marrow-matrix environment by expressing fibronectin, type IV collagen, and laminin. Stem Cells.

[2] Malara A., Gruppi C., Celesti G., Abbonante V., Viarengo G., Laghi L., De Marco L., Muro A.F., Balduini A. (2018). Alternatively spliced fibronectin extra domain a is required for hemangiogenic recovery upon bone marrow chemotherapy. Haematologica.

[3] Malara A., Abbonante V., Di Buduo C.A., Tozzi L., Currao M., Balduini A. (2015). The secret life of a megakaryocyte: Emerging roles in bone marrow homeostasis control. Cell Mol. Life Sci..

[4] Gong Y., Zhao M., Yang W., Gao A., Yin X., Hu L., Wang X., Xu J., Hao S., Cheng T. (2018). Megakaryocyte-derived excessive transforming growth factor β1 inhibits proliferation of normal hematopoietic stem cells in acute myeloid leukemia. Exp. Hematol..

[5] Bruns I., Lucas D., Pinho S., Ahmed J., Lambert M.P., Kunisaki Y., Scheiermann C., Schiff L., Poncz M., Bergman A. (2014). Megakaryocytes regulate hematopoietic stem cell quiescence through cxcl4 secretion. Nat. Med..

[6] Zhao M., Perry J.M., Marshall H., Venkatraman A., Qian P., He X.C., Ahamed J., Li L. (2014). Megakaryocytes maintain homeostatic quiescence and promote post-injury regeneration of hematopoietic stem cells. Nat. Med..

[7] Abbonante V., Di Buduo C.A., Gruppi C., Malara A., Gianelli U., Celesti G., Anselmo A., Laghi L., Vercellino M., Visai L. (2016). Thrombopoietin/TGF-β1 loop regulates megakaryocyte extracellular matrix component synthesis. Stem Cells.

[8] Malara A., Gruppi C., Rebuzzini P., Visai L., Perotti C., Moratti R., Balduini C., Tira M.E., Balduini A. (2011). Megakaryocyte-matrix interaction within bone marrow: New roles for fibronectin and factor xiii-a. Blood.

[9] Abbonante V., Chitalia V., Rosti V., Leiva O., Matsuura S., Balduini A., Ravid K. (2017). Upregulation of lysyl oxidase and adhesion to collagen of human megakaryocytes and platelets in primary myelofibrosis. Blood.

[10] Machlus K.R., Italiano J.E. (2013). The incredible journey: From megakaryocyte development to platelet formation. J. Cell Biol..

[11] Krishnaswamy V.R., Mintz D., Sagi I. (2017). Matrix metalloproteinases: The sculptors of chronic cutaneous wounds. Biochim. Biophys. Acta.

[12] Rundhaug J.E. (2005). Matrix metalloproteinases and angiogenesis. J. Cell Mol. Med..

[13] Mannello F., Tonti G.A., Bagnara G.P., Papa S. (2006). Role and function of matrix metalloproteinases in the differentiation and biological characterization of mesenchymal stem cells. Stem Cells.

[14] Lane W.J., Dias S., Hattori K., Heissig B., Choy M., Rabbany S.Y., Wood J., Moore M.A., Rafii S. (2000). Stromal-derived factor 1-induced megakaryocyte migration and platelet production is dependent on matrix metalloproteinases. Blood.

[15] Choi W.S., Jeon O.H., Kim H.H., Kim D.S. (2008). Mmp-2 regulates human platelet activation by interacting with integrin alphaiibbeta3. J. Thromb. Haemost..

[16] Gresele P., Falcinelli E., Loffredo F., Cimmino G., Corazzi T., Forte L., Guglielmini G., Momi S., Golino P. (2011). Platelets release matrix metalloproteinase-2 in the coronary circulation of patients with acute coronary syndromes: Possible role in sustained platelet activation. Eur. Heart J..

[17] Guglielmini G., Appolloni V., Momi S., De Groot P.G., Battiston M., De Marco L., Falcinelli E., Gresele P. (2016). Matrix metalloproteinase-2 enhances platelet deposition on collagen under flow conditions. J. Thromb. Haemost..

[18] Wrzyszcz A., Wozniak M. (2012). On the origin of matrix metalloproteinase-2 and -9 in blood platelets. Platelets.

[19] Mannello F., Medda V. (2011). Differential expression of MMP-2 and MMP-9 activity in megakaryocytes and platelets. Blood.

[20] Villeneuve J., Block A., Le Bousse-Kerdiles M.C., Lepreux S., Nurden P., Ripoche J., Nurden A.T. (2009). Tissue inhibitors of matrix metalloproteinases in platelets and megakaryocytes: A novel organization for these secreted proteins. Exp. Hematol..

[21] Cecchetti L., Tolley N.D., Michetti N., Bury L., Weyrich A.S., Gresele P. (2011). Megakaryocytes differentially sort mrnas for matrix metalloproteinases and their inhibitors into platelets: A mechanism for regulating synthetic events. Blood.

[22] McCawley L.J., Matrisian L.M. (2001). Matrix metalloproteinases: They’re not just for matrix anymore!. Curr. Opin. Cell Biol..

[23] Jobin P.G., Butler G.S., Overall C.M. (2017). New intracellular activities of matrix metalloproteinases shine in the moonlight. Biochim. Biophys. Acta.

[24] Ip Y.C., Cheung S.T., Fan S.T. (2007). Atypical localization of membrane type 1-matrix metalloproteinase in the nucleus is associated with aggressive features of hepatocellular carcinoma. Mol. Carcinog..

[25] Mohammad G., Kowluru R.A. (2011). Novel role of mitochondrial matrix metalloproteinase-2 in the development of diabetic retinopathy. Invest. Ophthalmol. Vis. Sci..

[26] Mannello F., Medda V. (2012). Nuclear localization of matrix metalloproteinases. Prog. Histochem. Cytochem..

[27] Aldonyte R., Brantly M., Block E., Patel J., Zhang J. (2009). Nuclear localization of active matrix metalloproteinase-2 in cigarette smoke-exposed apoptotic endothelial cells. Exp. Lung Res..

[28] Kwan J.A., Schulze C.J., Wang W., Leon H., Sariahmetoglu M., Sung M., Sawicka J., Sims D.E., Sawicki G., Schulz R. (2004). Matrix metalloproteinase-2 (MMP-2) is present in the nucleus of cardiac myocytes and is capable of cleaving poly (adp-ribose) polymerase (parp) in vitro. FASEB J..

[29] Si-Tayeb K., Monvoisin A., Mazzocco C., Lepreux S., Decossas M., Cubel G., Taras D., Blanc J.F., Robinson D.R., Rosenbaum J. (2006). Matrix metalloproteinase 3 is present in the cell nucleus and is involved in apoptosis. Am. J. Pathol..

[30] Eguchi T., Kubota S., Kawata K., Mukudai Y., Uehara J., Ohgawara T., Ibaragi S., Sasaki A., Kuboki T., Takigawa M. (2008). Novel transcription-factor-like function of human matrix metalloproteinase 3 regulating the ctgf/ccn2 gene. Mol. Cell. Biol..

[31] Mannello F., Luchetti F., Canonico B., Falcieri E., Papa S. (2004). Measurements, zymographic analysis, and characterization of matrix metalloproteinase-2 and -9 in healthy human umbilical cord blood. Clin. Chem..

[32] Suzuki K., Bose P., Leong-Quong R.Y., Fujita D.J., Riabowol K. (2010). Reap: A two minute cell fractionation method. BMC Res. Notes.

[33] Ligi D., Mosti G., Croce L., Raffetto J.D., Mannello F. (2016). Chronic venous disease—Part II: Proteolytic biomarkers in wound healing. Biochim. Biophys. Acta.

[34] Tschesche H., Zölzer V., Triebel S., Bartsch S. (2001). The human neutrophil lipocalin supports the allosteric activation of matrix metalloproteinases. Eur. J. Biochem..

[35] Shirvaikar N., Reca R., Jalili A., Marquez-Curtis L., Lee S.F., Ratajczak M.Z., Janowska-Wieczorek A. (2008). Cfu-megakaryocytic progenitors expanded ex vivo from cord blood maintain their in vitro homing potential and express matrix metalloproteinases. Cytotherapy.

[36] Wilson C.L., Matrisian L.M. (1996). Matrilysin: An epithelial matrix metalloproteinase with potentially novel functions. Int. J. Biochem. Cell Biol..

[37] Dunsmore S.E., Saarialho-Kere U.K., Roby J.D., Wilson C.L., Matrisian L.M., Welgus H.G., Parks W.C. (1998). Matrilysin expression and function in airway epithelium. J. Clin. Invest..

[38] Li Q., Park P.W., Wilson C.L., Parks W.C. (2002). Matrilysin shedding of syndecan-1 regulates chemokine mobilization and transepithelial efflux of neutrophils in acute lung injury. Cell.

[39] Qu P., Yan C., Du H. (2011). Matrix metalloproteinase 12 overexpression in myeloid lineage cells plays a key role in modulating myelopoiesis, immune suppression, and lung tumorigenesis. Blood.

[40] Wang J., Ye Y., Wei G., Hu W., Li L., Lu S., Meng Z. (2017). Matrix metalloproteinase12 facilitated platelet activation by shedding carcinoembryonic antigen related cell adhesion molecule1. Biochem. Biophys. Res. Commun..

[41] Kile B.T. (2014). The role of apoptosis in megakaryocytes and platelets. Br. J. Haematol..

[42] Patel S.R., Hartwig J.H., Italiano J.E. (2005). The biogenesis of platelets from megakaryocyte proplatelets. J. Clin. Investig..

